# Does prediction error during exposure relate to clinical outcomes in cognitive behavior therapy for social anxiety disorder? A study protocol

**DOI:** 10.3389/fpsyt.2022.1000686

**Published:** 2022-11-24

**Authors:** Christopher D. Winkler, Peter Koval, Lisa J. Phillips, Kim L. Felmingham

**Affiliations:** Melbourne School of Psychological Sciences, University of Melbourne, Melbourne, VIC, Australia

**Keywords:** anxiety, prediction error, expectancy violation, exposure therapy, social anxiety disorder, fear, extinction

## Abstract

Facing your fears, or exposure therapy, is an effective psychological intervention for anxiety disorders that is often thought to work through fear extinction learning. Fear extinction learning is a type of associative learning where fear reduces through repeated encounters with a feared situation or stimulus in the absence of aversive outcomes. Laboratory research suggests fear extinction learning is driven by threat prediction errors, defined as when fearful predictions do not eventuate. Threat prediction error and its relationship to exposure therapy outcomes haven’t been studied enough in actual therapy settings. It remains unclear whether prediction error and extinction learning are central mechanisms of exposure therapy. We are conducting a longitudinal and observational study of how threat prediction error during exposure in social anxiety disorder (SAD) treatment relates to session-by-session symptom change and treatment outcome in addition to exposure surprise and learning outcome. We aim to recruit 65 adults with a primary diagnosis of SAD through an outpatient psychology clinic. Participants will receive 12 sessions of individual manualized cognitive behavioral therapy (CBT), adapted from an efficacious group protocol, that includes graded exposure. Exposure processes, including self-report measures of anxiety, threat prediction, threat outcomes, surprise, and learning outcome, will be measured with smartphone-based event-contingent ecological momentary assessments (EMAs) of all behavioral experiments completed during treatment. Clinical outcomes include self-reported social anxiety symptoms and social threat appraisals, at each session, post and 3-months after treatment. Prediction error will be operationalized as the mismatch between the threat prediction and threat outcome. The joint effect of threat prediction and threat outcome on session-by-session symptom change, treatment outcome, exposure surprise, and learning outcome will be explored using multilevel modeling. The present study will help determine whether threat prediction error during exposures in SAD treatment is related to theoretically implied clinical outcomes. This would contribute to the larger research aim of clarifying exposure therapy mechanisms.

## Introduction

Anxiety disorders are defined by anxiety, fear, and avoidance that impacts significantly negatively on functioning (1). They are the most common type of mental disorder worldwide (2), a leading cause of disability (3), and one of the most costly mental disorders in Australia (4). Cognitive behavioral therapy (CBT) effectively treats anxiety disorders (5–7) and is widely adopted in treatment guidelines [e.g., (8–10)]. Although CBT is effective for many people, around half do not respond adequately (11).

A central component of CBT for anxiety disorders is exposure; therefore, research to improve exposure-based interventions would potentially improve treatment outcomes. Exposure therapy involves repeatedly and systematically approaching feared but safe situations (12). The theory underlying the mechanisms of exposure therapy and best practice has changed over time (13). For decades, a dominant model was Emotional Processing Theory (EPT) (14) which argued that exposure worked by activating pathological “fear structures” in memory and then updating memory with corrective information that is incompatible with the pathological aspects of the structure. A fear structure was defined as a network of information in memory about the feared stimulus, appraisal of its threat and meaning, and responses to the stimulus. These structures are excessive and pathological in anxiety disorders (14–16). Emotional processing referred to when fear structures are updated with corrective information from exposure, leading to reduced threat appraisal and fear responding (14, 17). Foa and Kozak originally argued that fear activation, within exposure fear reduction (termed “within session habituation”; WSH), and between exposure fear reduction (termed “between session habituation”; BSH) were all indicators of emotional processing and therefore will predict better treatment outcome. Accordingly, exposure therapists designed and implemented exposures to achieve fear reduction by teaching patients to remain exposed until their fear reduced. However, many treatment studies have found that fear reduction within exposure sessions didn’t reliably predict treatment outcome and wasn’t necessary for corrective learning (18–20). This challenged the importance of within exposure fear reduction.

Noting the lack of evidence for within exposure fear reduction as a mechanism of exposure, Craske and colleagues drew from contemporary learning theory, supported by decades of laboratory research in animals and humans, to propose an updated model of exposure. The inhibitory retrieval model (12, 18, 21, 22) proposes that exposure works through fear extinction learning mechanisms. Fear extinction learning is a type of associative learning, typically studied in the laboratory, where fear reduces through repeated encounters with a feared situation or stimulus in the absence of aversive outcomes (23). On encountering the feared stimuli, relevant threat information is retrieved from memory in the form of learned associations between the stimulus and the presence of aversive outcomes. The brain uses this information to make predictions about the likelihood and severity of aversive outcomes during the encounter (12, 24). New learning is thought to occur if the brain detects a prediction error, defined as an unexpected event (24–27). The amount of prediction error determines the strength of new learning, while the direction determines the type of new learning (24, 25, 27). It is widely agreed that prediction error is closely related to surprise (24, 28). The cognitive-evolutionary model of surprise (29, 30) proposes that surprise is a universal emotion that serves to motivate analysis of unexpected events and to facilitate learning. This model proposes that the brain monitors for unexpected events, and if detected a signal is generated that codes the degree and type of prediction error experienced. This prediction error signal is experienced consciously as the feeling of surprise, should it pass a certain threshold (30). From an evolutionary perspective, prediction error processing and surprise mechanisms enable the brain to more accurately model a changing world and thus increase the chances of survival (30). Support for this model comes from non-clinical surprise research [for a review see, (30)], which found that unexpected events induce self-reported surprise [e.g., (31, 32)] with surprise intensity being caused by the degree of event unexpectedness [e.g., (33, 34)].

Fear extinction is thought to occur following negative threat prediction errors, defined as when the rate or severity of aversive outcomes is *less than expected* during an encounter (21, 24, 25, 27). Conversely, fear learning is thought to occur following positive threat prediction errors, defined as when the rate or severity of aversive outcomes is *greater than expected* during an encounter (24, 25). Extinction learning is an inhibitory process where new memories are formed to represent the safety of the stimulus rather than old threat associations being “unlearned” (35). New safety associations that are acquired through extinction training compete with prior threat learning for retrieval when a person encounters relevant cues (35). Additionally, new safety associations are linked to their learning context and may not generalize or be retrieved if the stimulus is encountered in a different context (35). Extinction learning being inhibitory and context dependent can explain how, after a course of successful extinction training, fear can return in new contexts or spontaneously after a period of time, and also can return rapidly after subsequent aversive experiences (35).

Craske et al. (12, 18, 21, 22) translated the above inhibitory learning/retrieval model to exposure therapy to derive recommendations for therapists to optimize treatment effects. In this model, threat prediction error (rather than fear reduction) drives corrective learning so that an exposure will only be therapeutic to the extent that the patient’s fears don’t come true (a negative prediction error). The degree of negative prediction error is thought to determine the amount of corrective learning so therapists should conduct exposures in a way that achieves maximal negative prediction error (rather than fear reduction *per se*). Strategies to maximize negative prediction error included eliciting threat patient predictions and perceptions of the outcome, designing exposures to enable proper testing of threat predictions, encouraging patients to drop subtle avoidances during exposures, and sequencing exposures so they are more unpredictable. The inhibitory learning/retrieval model of exposure also implied that relapse (i.e., return of fear) may be prevented by exposure practice that is varied in context and well consolidated in memory.

Foa et al. updated EPT to incorporate the inhibitory extinction learning model and adjust for the evidence that WSH had not reliably predicted treatment response (36, 37). Accordingly, EPT now holds that corrective information from exposures is encoded into new fear structures in memory that are non-pathological and that compete with old pathological fear structures. EPT now also de-emphasizes WSH and rather focuses on prediction error as the mechanism that drives corrective learning from exposure. EPT differs from the inhibitory learning/retrieval model in its concept of fear structures that include not only associations but also beliefs and higher order cognitions. According to EPT, prediction errors may create new associative memories but also lead to new meanings and beliefs that then reduce fear (37). Aside from this point of difference, both the inhibitory learning/retrieval model and EPT propose that exposure therapy operates through extinction learning mechanisms and prediction error processing.

Extinction learning mechanisms outlined above are supported by laboratory research and, when applied to exposure, can explain why fear reduction during exposure had not reliably predicted treatment outcomes in past research. Alongside the above theoretical developments, Benito et al. (38) recently raised the alternative possibility WSH had not reliably predicted treatment outcomes in previous research because of measurement problems. Past research typically calculated a WSH index by subtracting the subjective fear rating at the end of an exposure from the peak fear rating during the exposure, missing potentially important fear fluctuations during the exposure (38). Benito et al. (38) also argued that past exposure research had not adequately controlled for confounding non-learning factors that could cause fear reductions during exposure. Benito et al. (38) used a novel fear reduction metric for WSH during exposure and found that it predicted treatment outcomes for youth with OCD. Benito et al. (38) used observers to continuously rate patient fear and other contextual events during exposures in treatment. WSH was defined as the sum of all fear reductions during an exposure (rather than peak minus end) while parsing out fear reductions due to confounding variables (e.g., escape or use of rituals).

The recent work of Bonito et al. (38) demonstrates that WSH may yet be a key marker of corrective learning and further research is needed to clarify. Regardless, the inhibitory retrieval model of exposure, including prediction error-driven learning, has been influential and adopted in contemporary treatment guides [e.g., (13)]. This model was derived from laboratory research with animals and humans and assumes that laboratory research translates to therapy settings (39). To date, there is a lack of research testing whether extinction learning mechanisms, including threat prediction error, account for “real world” exposure therapy effects and so its external validity as a treatment model isn’t yet established (39). Clinical treatment is substantially more complex than laboratory conditions and so it is possible that other processes contribute to exposure effects (40). Further research is needed to determine whether threat prediction error is necessary for exposure efficacy, and if so, how it relates to therapeutic change. Further research is needed to determine the relative importance of prediction error to other mechanisms of change. Answering these questions is important as therapists use theoretical models to guide their practice and explain their treatments to patients.

Only a handful of studies to date have explored whether prediction error during exposure relates to treatment outcomes. This hypothesis has been tested in exposure-based treatment for obsessive compulsive disorder (41–43), panic disorder (44), and post-traumatic stress disorder (45). Results have been mixed with some studies finding an association between prediction error during exposure and treatment outcomes (41, 43), while other studies have found no such effects (42, 45). These mixed results may be due to three limitations in the research—broadly described as conceptual, statistical, and methodological issues that are summarized below.

The first limitation relates to how threat prediction error should be measured and operationalized. A common practice is to operationalize threat prediction error as the algebraic difference score between two component variables measured by self-report. The first component assesses how much the patient is predicting aversive outcomes (threat prediction) and the second component assesses how much the patient thought these outcomes occurred during the exposure (threat outcome). Although simple to use, difference scores have well-known statistical and conceptual problems when exploring the joint effects of two component variables on an outcome variable, such as in the current case of prediction error to outcome (46, 47). Difference scores are less reliable than their component measures and can conceal true mismatch effects by artificially reducing what is a three-dimensional relationship to a two-dimensional relationship (47). For example, any observed relationship found between a difference score and therapy outcome could be attributed to threat prediction only and not threat outcome, or vice versa. Complex mismatch effects may also be possible, for example, prediction errors may be more impactful when the components are both at higher vs. lower absolute levels. Outside of clinical psychology, the best practice for exploring non-linear or complex mismatch or congruence effects is polynomial regression with response surface analysis (RSA) (47–51). The best practice for exploring linear discrepancy effects currently is condition-based regression analysis (CRA) (52), an approach related to RSA.

In CRA and RSA, the components of a difference score (e.g., threat prediction and threat outcome) are entered separately into the regression equation and a linear discrepancy effect is demonstrated if their regression coefficients are both significant and are opposite in sign (52). The presence of complex or non-linear mismatch effects can be explored by adding higher order terms and using resulting regression coefficients to plot a response surface, perform significance tests, and interpret response surface parameters (49–51, 53). To date, no studies of the relationship between threat prediction error and clinical outcomes in a therapy context have modeled threat prediction and outcome variables separately or considered the possibility of complex mismatch effects analytically.

The second limitation of extant research is that prediction error has not been statistically or conceptually separated into within- and between-person components. Therapy process-outcome research is usually concerned with within-person processes (54, 55). How prediction errors experienced by an individual relate to changes in that individual’s anxiety over time is a within-person effect. By contrast, a between-person effect may be that of individual differences in the trait propensity to experience prediction error on treatment outcome. Research to date has typically averaged the difference scores between predictions and outcomes across all exposures in treatment. Such averages comprise a mix of within- and between-person information, confounding their interpretation (54). Furthermore, effects at the within- and between-person levels can differ in magnitude and direction and so should be separated conceptually and statistically to prevent confounds (54, 56).

The third limitation of existing research is that it has typically examined in-session exposures only, meaning data from homework exposures completed by the patient between sessions were not considered. Patients are typically encouraged to do exposures outside of sessions and these may be even more meaningful since they are done without the support of the therapist. A complete investigation of the prediction error hypothesis would thus benefit from all exposures in treatment being examined.

Finally, exposure therapy mechanism research has not (to the author’s knowledge) explicitly explored the relationship between expectations, prediction error, and surprise. Theorists seem to agree that these constructs are closely related (24, 28, 29) and non-clinical research suggests that the conscious feeling of surprise is caused by unexpected events (30).

The goal of this study is to determine the sequelae of prediction error (both amount and type) during exposures on clinical outcomes in CBT for social anxiety disorder (SAD). This disorder is characterized by excessive fear and avoidance of social situations (57). CBT for SAD presents a good case for exploring exposure prediction error effects for several reasons. First, SAD is one of the most common anxiety disorders (58), meaning recruitment may be easier and any results will be pertinent to many therapists and patients. Second, CBT is widely used and efficacious for SAD (59). Third, evidence-based CBT treatments have exposure as a core treatment component in the form of a systematic approach toward feared social situations (60–64). Finally, at the time of study design, prediction error had not been studied explicitly in CBT for SAD. Our study has three aims:

(1) The primary aim of the current study is to explore whether threat prediction error experienced during exposures in and between sessions is related to session-by-session symptom changes. We will model the effects of threat prediction and outcomes separately rather than using difference scores while isolating within-person from between-person effects, with the former being of primary interest. We will sample both in-session and homework exposures in treatment by using smart-phone based ecological momentary assessment (EMA) and generate rich descriptive information about patient exposure experiences. We hypothesize that prediction errors will be common in treatment and that negative threat prediction error during exposures between sessions will predict symptom reduction between sessions. We hypothesize that positive prediction error during exposures between sessions will predict symptom increase between sessions.

(2) A secondary aim of the current study is to explore whether the average magnitude of prediction errors experienced during treatment is associated with treatment outcome. We hypothesize that patients who experienced a greater mean level of negative prediction error across all exposures in treatment will report fewer symptoms at the end of treatment and after 3 months.

(3) A tertiary aim will be to explore the construct validity of using self-reported threat prediction and outcome to measure prediction error. Prediction error is thought to relate to learning outcomes and generate feelings of surprise and surprise is positively related to prediction error in non-clinical research (30), so we hypothesize that exposures with greater prediction error, regardless of type, will be more surprising to the patient and the patient will be more likely to report having learned something from it. To our knowledge, no study to date has examined the above in anxiety disorder treatment.

## Methods and analysis

### Design

This longitudinal and observational study will be embedded in a larger project evaluating predictors and moderators of symptom change in adults receiving individual CBT for SAD. This treatment includes graded exposure-based behavioral experiments conducted in sessions and for homework. Assessments will be made at baseline, before each session, mid-treatment, post-treatment, and 3-month follow-up and only those relevant to the current study will be described (see Table 1). Exposure processes are the predictors of interest for this study and will be measured using event-contingent EMA of all exposures completed during treatment.

**TABLE 1:** Study assessment schedule.

Measure	Assessment point															
	B	1	2	3	4	5	6	M	7	8	9	10	11	12	P	3M
SIPS	X*	X	X	X	X	X	X	X*	X	X	X	X	X	X	X*	X*
STATM	X	X	X	X	X	X	X	X	X	X	X	X	X	X	X	X
EPQ																
In-session						X	S		V	X	X					
Event contingent						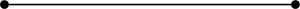										

B, Baseline; 1–12, Sessions 1–12; M, Mid treatment; P, Post treatment; 3M, 3 month follow up. SIPS, Social interaction phobia scale; STATM, Social threat appraisal tracking measure; EPQ, Exposure process questionnaire; S, Safety behavior experiment version; V, Video review experiment version. *SIPS extracted Social Interaction Anxiety Scale and Social Phobia Scale in the parent study.

### Recruitment

Recruitment and treatment will take place at the University of Melbourne Psychology Clinic (“the clinic”). The clinic offers low-cost psychological therapy for mental disorders and serves the local community. Clinical services are delivered by master’s level intern therapists completing their first placement in their post-graduate clinical psychology training. They are supervised by senior clinical psychologists. Therapists and their supervisors will be asked to offer study participation as a treatment option for all their eligible clients, following an initial assessment. Social anxiety is a common presenting issue at the clinic and community advertisements for the study will also be placed to help with recruitment. Interested clients will be contacted by a researcher to complete informed consent, study orientation, and assessment scheduling. Clinic therapists will diagnose mental disorders according to the Diagnostic and Statistical Manual of Mental Disorders [5 ed. DSM-V; (57)] using the Diagnostic Interview for Anxiety, Mood, and Obsessive-Compulsive and Related Neuropsychiatric Disorders (DIAMOND) (65) for all clients. Each year, the clinic takes on a new cohort of approximately 20 intern therapists as the previous year’s cohort concludes their placement.

To improve the recruitment rate, a researcher will inform each new therapist cohort of the study and ask them to recruit all eligible clients they assess on placement at the clinic. Each year, key parties (students, supervisors, and the clinic director) will be advised of the study and how to recruit by email. The first author will also conduct a 2-h information and orientation session for each starting therapist cohort on the study protocol and treatment manual. Participants will be remunerated with free study treatment, $30 vouchers for each completed baseline, mid-treatment, and post-treatment assessments (for a total of $90). In addition, they will be reimbursed $1 for each EMA survey they complete in treatment regarding an exposure.

### Eligibility

Adults (aged 18–70 years) with a primary DSM-5 diagnosis of SAD and no exclusion criteria may participate. Exclusion criteria are current severe substance use disorder, current severe major depressive episode (to a degree that precludes engagement in social anxiety therapy), acute suicide risk, current non-suicidal self-injury, current or history of psychotic illness or bipolar disorder, or cognitive or language difficulty that prevents engagement in CBT. Other co-morbidity is acceptable so long as SAD is primary. Co-morbidity will be included to ensure that the sample is like patients typically seen in community mental health settings, where co-morbidity is common (66). Eligibility will be decided by therapists in consultation with their supervisor supported by the DIAMOND. Decisions on whether depression, cognitive, or language difficulties are severe enough to prevent engagement in the treatment are made based on the judgment of the therapist and their supervisor considering the full range of assessment information. For depression, this would likely indicate that the depression was the primary diagnosis. The first author will be available for secondary consultation if either therapist or supervisor is unsure.

### Treatment

Therapists will deliver 12 sessions of individual manualized CBT for SAD delivered in person or by video teleconference (if the participant prefers or should local COVID-19 regulations preclude the former). Sessions are 60 min in duration, except for sessions six and nine which are 90 min to allow for more time to do in session exposures. Sessions are intended to occur on a weekly basis where possible but may be compressed or spaced if necessary for practical reasons or clinical needs, as decided by the patient and their therapist/supervisor.

We obtained permission to adapt an existing group therapy protocol (67) to an individual format that incorporated smartphone-based recording of exposures (vs. pen-paper worksheets) for use in the current study. Group-to-individual format adaptations were made by the first author with reference to other published individual SAD treatment manuals (60–62) and to preserve the same treatment components and sequencing as the original. The first author also added guidance for applying the treatment over video teleconference and during pandemic conditions. In-person and video teleconference content was written to preserve as much consistency in these modes as possible. The original group protocol effectively treats SAD (63, 68) with interventions designed to modify SAD-specific maintenance processes (69, 70). Components include psychoeducation about social anxiety, cognitive restructuring of social threat appraisals and negative core beliefs, graded exposure with a threat-prediction testing rationale, weaning of safety behaviors, attentional focus training, correcting negative self-perception with objective feedback, and relapse/maintenance planning (63).

#### Exposure interventions

Exposure is a central feature of the planned intervention. Participants develop one or more hierarchies of feared/avoided social situations and are then supported to systematically approach these while dropping subtle avoidance and anxiety-driven coping strategies called *“safety behaviors.”* These exposures are framed as behavioral experiments with a threat prediction testing rationale rather than a habituation rationale. Exposures begin from session 5 and are set for homework until the end of treatment. In session 5, the participant and therapist develop one or more graded hierarchies of feared social situations (depending on the needs of the participant) to work through in treatment. Participants are encouraged to do exposures from their hierarchies for homework from session 5 onward.

All exposures are determined collaboratively by the therapist and participant within the constraint that it targets the participant’s social threat cognition and associated social situation. Exposures will thus differ for each participant since they are determined based on individual needs. Examples of possible exposures include initiating conversation, talking in group conversations, dating, being assertive, talking to authority figures, eating in public, public speaking, dancing in public, or talking on the phone. The number of exposures completed for homework will likely vary for each participant, depending for example on participant motivation and compliance. Since the study will operate in pandemic conditions, homework exposures will adhere to any social distancing or lockdown regulations at the time.

In-session exposures that are guided by the therapist occur in sessions 5, 6, 7, 8, and 9. The session 5 exposure aims to introduce doing exposure using smartphone EMA while providing an early positive experience. Therapists and participants can choose the specific social activity, but the manual recommends choosing one that can be practical to complete in-session, relevant to the participant, achievable for the participant, but also that elicits anxiety. Examples could include giving a short speech or having a short social interaction. This could be with the therapist, people in the clinic, or people in short walking distance from the clinic.

The session 6 exposure aims to help the participant evaluate the utility of safety behaviors in social situations. Participants complete a social activity twice, once using safety behaviors and once without, to challenge their fears about dropping safety behaviors and explore the impact of using them in social situations [originally from Clark et al.’s cognitive therapy, (71)]. Therapists are instructed to choose a social activity that takes no longer than 5 min, to make the experiment practical to complete in a timely manner. Examples may include the participant interacting with the therapist (role play) or person from around the clinic.

The session 7 exposure aims to correct participant’s negative self-perception. The participant delivers a recorded speech and then reviews how they appeared compared to what they expected [similar to the procedure in Harvey et al. (72)]. Therapists may tailor the experiment to participants by adjusting the difficulty (for example, choosing a less familiar topic, arranging more audience members, or affording less preparation time).

The session 8 exposure aims to give participants practice focusing their attention on the task during social situations, rather than on the self or what the other person is thinking. Therapists are advised to choose a social interaction activity such as a role-play conversation with a therapist or colleague or making small talk with someone near the clinic. In-session exposures for sessions 5, 6, 7, and 8 can be conducted over video teleconference through role plays with the therapist or inviting confederates to the video call as needed.

Session 9 is devoted entirely to guided exposure where the participant attempts various exposures outside of the clinic with the support of their therapist. These exposures focus on the participant deliberately doing socially “odd” or notable behaviors to see what happens compared to their fears. Participants are supported to choose two exposures to complete with the therapist and two to complete individually during the session. A list of choices is provided but bespoke exposures can be developed in collaboration with the participant. An example of a paired exposure is the therapist and participant looking upwards and pointing for 5 min. An example of an individual exposure is to ask a shop assistant where an item is while standing in front of it. This session can be facilitated by the therapist over the phone or video teleconference as needed, where the participant completes the above exposures *in situ* with the therapist “in their pocket.”

### Outcomes

The primary outcome will be self-reported social anxiety severity as measured using the 14-item Social Interaction Phobia Scale (SIPS) (73). The SIPS was reduced from the longer SIAS (74) and SPS (74). The SIPS retains good psychometric properties whilst reducing participant burden and is suitable for session-by-session symptom tracking (73). The SIPS will be administered at 16 time points including before treatment, at each session, mid-treatment (between sessions 6 and 7), post-treatment, and 3 months after treatment.

Threat prediction error is thought to trigger extinction learning and correction of inflated threat appraisal (social threat in the case of social anxiety) (22, 36). Social threat appraisals will be the secondary outcome and will be measured 16 times on the same schedule as the primary outcome. Social threat appraisals will be measured using an instrument adapted by the first author for the purpose of this study named the Social Threat Appraisal Tracking Measure (STATM). The STATM was adapted from a similar measure developed by Gregory and colleagues (75) to briefly measure and track session-by-session changes in social threat appraisals during CBT treatment for SAD. Participants read two hypothetical social tasks involving public speaking and social interaction, respectively. For each task, they are asked if they would have any fears about attempting these tasks today. If yes, they are instructed to write down their feared outcomes in an open-ended format. Then, participants rate their anxiety, perceived likelihood of feared outcome, and perceived cost of their feared outcome if they were to attempt the task today, using slider scales anchored at 0 (*Not at all*) and 100 (*Extremely*). The mean of likelihood and cost ratings across both social tasks (i.e., four items) estimates the overall degree to which the participant views social situations as threatening at that time. Higher scores indicate a greater perception of social threat. If a participant says that they would not have any fears about attempting a task that day, their score will be coded as 0. To make score comparisons meaningful, the hypothetical social tasks in the measure remain the same over repeat administrations.

### Ecological momentary assessment procedure and measures

Prior to session five, a researcher will help each participant setup their smartphone to complete event-contingent EMA surveys *via* Qualtrics (76). Participants place re-usable hyperlinks on their smartphone home page for the duration of the study, which they can click on to initiate a survey in their web-browser. Participants also receive a user guide for their reference. Participants complete their first survey and exposure during session 5, guided by their therapist. For the remainder of treatment, therapists assign exposures for homework with verbal and written instructions for participants to complete a smartphone survey for each one. Therapists guide participants to complete surveys for additional in-session exposures during sessions 6, 7, 8, and 9.

We developed the EMA questionnaire for the purpose of this study, which we call the exposure process questionnaire. We designed the questionnaire to be clinically useful while still assessing variables of interest. It is an expanded digital version of the typical static pen-paper (or pdf) exposure record commonly used in CBT. The expanded items were mostly adapted from existing validated measures. The questionnaire has a pre- and post-experiment section and uses conditional branching to display relevant questions based on participants’ responses. Item response types include open-ended text fields, slider ratings, single-choice questions, and multiple-choice questions.

There are three versions of the exposure process questionnaire that share a core set of items (see Supplementary material for full description). The standard exposure process questionnaire (EPQ) is used for all in-session and homework exposures except for the safety behaviors exposure in session 6 and video review exposure in session 7. The EPQ assesses anxiety, threat predictions, threat outcomes, surprise, safety behavior use, learning outcome, and contextual features of the exposure (described in more detail below).

The exposure process questionnaire-safety behavior (EPQ-SB) contains the same core set of items as the EPQ, but with additional questions to help therapist-clients to explore the utility of safety behaviors during the exposure. Similarly, the exposure process questionnaire-video review (EPQ-VR) contains the same core set of items as the other surveys, but with additional questions to help therapist-clients to review how visible anxious symptoms were in a video recording of them performing a social exposure. We will merge data from the shared items on the EPQ, EPQ-SB, and EPQ-VR for the final analysis.

The pre-exposure section of the EPQ contains 11 fixed questions ending with a prompt to attempt the exposure and record its completion as soon as possible afterward. If the participant indicates that they completed the exposure, they are shown the post-exposure section. This section comprises 16 more fixed questions and 3 supplemental questions depending on fixed item responses. The survey ends and responses are recorded if participants indicate that they didn’t complete the exposure. Survey time stamps, item response times, and overall duration are recorded automatically.

For in-session exposures, the therapist will prompt the participant to start a new survey just before doing the exposure. The therapist will ask the participant to read out each of their responses to the pre-experiment section so that the therapist is aware of their ratings and threat predictions. Once the pre-experiment section is complete, the therapist will prompt the participant to complete the planned activity and then the post-experiment section of the survey immediately after. The therapist asks the participant to read out their post-experiment survey responses and then reviews actual vs. predicted outcomes with the participant and what they learned from the activity. In this way, the EPQ also functions as a clinical tool for the therapist to elicit threat cognitions, structure the exposure, and conduct cognitive review afterward. A new survey is initiated for each new exposure attempted in a session. For between-session exposures, participants will be instructed to start a new survey immediately before every attempted behavioral experiment. Participants will be instructed to complete the post-experiment section as soon as possible after attempting these experiments. Accordingly, participants will work through their survey responses themselves without pre-post discussion *in situ* with their therapist.

The questionnaire measures the following exposure variables.

#### Anxiety

Momentary anxiety intensity will be assessed before and after exposures through single slider ratings of *“How anxious do you feel right now?”* from 0 = *“Not at all”* to 100 = *“Extremely.”* Peak and end anxiety intensity during the exposure will be assessed retrospectively in the post-experiment section with slider ratings of *“How anxious were you at your peak level during the activity?”* and *“How anxious did you feel at the end of the activity?”* using the same anchors as the momentary ratings. Single-item anxiety rating scales have acceptable psychometric qualities, are suitable for quick administration (77, 78), and have been used in other exposure mechanism studies [e.g., (79)].

#### Threat prediction

We operationalize threat prediction (P) as the belief strength that negative outcomes will occur during a planned exposure. In the pre-exposure section, participants type what their worst fears are about doing the planned exposure into an open text field. They will then be asked to rate how strongly they believe four different outcomes will happen during the experiment using a slider scale from 0 = *“Not at all”* to 100 = *“Extremely.”* This first item, *“How strongly do you believe that your worst fears will happen during the activity?”* is worded so that it links to the idiosyncratic threat cognition stated in the previous open-test question. The remaining three items assess common concerns of those with SAD and were “*How strongly do you believe that you will be judged negatively during the activity?”* “*How strongly do you believe that you will make a bad impression during the activity?”* and “*How strongly do you believe that you will appear anxious during the activity?”* These items were adapted from established social anxiety measures in the literature (80–83) to represent broad themes suggested by scale factor structures and preferencing higher loading items. The items were worded broadly to apply to any social anxiety-specific exposure. The overall expected aversiveness of a specified exposure will be assessed with a single slider rating of *“How bad do you think the outcome of the activity will be?”* on a scale of 0 = *“Not at all”* to 100 = *“Extremely.”* For analysis, the mean of all belief strength ratings will be used to assess overall threat prediction for the specified exposure.

#### Threat outcome

Perception of threat outcome during a planned exposure is operationalized as the belief strength that predictions occurred during the exposure. Threat outcome (O) will be measured using parallel versions of the four O items which share the same scale (slider rating of strength belief from 0 = *“Not at all”* to 100 = “*Extremely*”) and content dimension (occurrence of threat), but different reference points (predicted vs. experienced). For example, the P item *“How strongly do you believe that your worst fears will happen during the activity?”* corresponds with the O item *“How strongly did you believe that your worst fears happened during the activity?”* Commensurate measurement of P and O enables meaningful interpretation of any joint effects they have on the outcome variables (47). The mean of belief strength ratings for the four O items will be used to assess the overall threat outcome for the specified exposure. The overall perceived actual aversiveness of a specified exposure will be assessed with a commensurate version of the expected aversiveness item in the pre-exposure section (i.e., *“How bad do you think the outcome of the activity was?”* on a scale of 0 = *“Not at all”* to 100 = *“Extremely”)*.

#### Surprise

Surprise about the outcome of an exposure will be assessed in the post-exposure section with a single item *“How surprised do you feel about the outcome of the activity?”* rated on a slider scale from 0 = *“Not at all”* to 100 = *“Extremely.”* We chose a single-item measure for surprise to keep the EMA questionnaire short given that surprise related to a tertiary study aim and data quality suffers with longer questionnaires in experience sampling studies (84). Furthermore, surprise researchers have often measured the feeling of surprise with a single rating, measure of surprise intensity, which has shown to respond to unexpected events and degree of event unexpectedness (32–34). The “surprised” item also loads strongly on the three-item surprise scale of the Positive and Negative Affect Scale—Expanded Form (amazed and astonished being other items) which is a reliable and validated measure of affective states (85).

#### Safety behaviors

Participants will rate how much they used any safety behaviors to manage their anxiety during the exposure on a slider rating scale from 0 = “*Not at all”* to 100 = *“As much as possible.”* Participants view a definition of safety behaviors when making this rating, that they are *“things people do sometimes in social situations to manage their anxiety, such as avoiding eye contact, remaining very quiet, making an effort to get your words right, trying to not look anxious (e.g., trying not to shake sweat, blush) or rehearsing sentences in your mind.”* Similar rating scales have been used in social anxiety safety behaviors research and have evidence for clinical utility and validity (86, 87).

#### Learning outcome

In the post-exposure section, participants indicate whether they learned anything from the exposure (single choice *“yes,” “no,” “not sure”*). If they select *“yes,”* they are prompted to type what they learned (open-ended text field).

#### Contextual features

In the pre-exposure section, participants type what social activity they plan to do and indicate whether they have attempted this activity before and if it is occurring in or between session. In the post-exposure section, participants indicate how soon after the exposure they are completing the questions, how long the exposure took, what the actual outcome of the exposure was, the type of location the exposure took place in, and the type of interaction partners present.

### Fidelity

Therapists and their supervisors will be asked to adhere to a detailed treatment manual containing session plans, handouts, worksheets, and therapist guidance. The first author will be available for *ad hoc* secondary consultation and advice on applying the treatment and to resolve any technical problems with smartphone surveys. Therapist competence will not be assessed in the study protocol, but therapists are required to attend weekly individual supervision in their clinic placement. Adherence will not be assessed directly, but therapists will be instructed to complete a tracking survey after each session to record any significant deviations from the manual. A researcher will monitor and respond to data quality concerns but will not have access to the clinical record. Participant compliance and exposure dose will be indicated by the number of exposures recorded by the participant using smartphone EMA. Participants will be asked each session whether they didn’t record any exposures using their smartphone, and if so, how many.

### Analysis

The relationship between exposure processes and outcome variables over time will be explored using univariate multilevel modeling (MLM). This modeling framework has a range of strengths for our purposes. It can account for data dependencies typical in longitudinal designs, can handle assessment schedules that are unevenly spaced, and can handle unbalanced data (88). Prediction error is the exposure process of interest. We operationalize prediction error as the discrepancy between threat prediction (P) and threat outcome (O) scores for an exposure.

Prediction error effects can only be explored if enough cases of prediction error exist in the data. We will explore how many cases of prediction error and what type are present in the data before modeling. For descriptive purposes, we will categorize a prediction error as an exposure where the standardized P and O scores differ by more than 0.5 standard deviation in either direction (89). We will proceed with modeling prediction error effects if more than 10% of exposures meet the above prediction error criteria (49).

The primary aim of the study is to explore how prediction error during exposures relates to session-by-session symptom change. Exposures may occur from sessions 5 to 12. The outcomes are measured once at each session, but the number of exposures completed may vary significantly over treatment. We will aggregate EMA data obtained between sessions (e.g., between sessions 5 and 6, or 6 and 7, etc.) to form seven time points. Symptoms at time *t* will be regressed onto the mean P and mean O in the preceding inter-session interval (i.e., between *t* – 1 and *t*). We will control for the effect of symptoms at the preceding session (*t* – 1) and other theoretically relevant exposure mechanisms, specifically mean fear activation (operationalized as mean peak anxiety minus mean pre anxiety) and mean within-exposure habituation (operationalized as mean peak anxiety minus mean end anxiety) in the preceding inter-session interval. Operationalizing fear activation and habituation in these ways is consistent with most prior research, but we acknowledge the limitations of these indices noted by Bonito et al. (38) and will discuss the results in light of these.

In line with learning theory, we hypothesize that people whose exposures since the last session (*t* – 1) went better than expected will have lower social anxiety symptoms and social threat appraisals at the next session (*t*). Specifically, when holding mean O and other controls constant, higher mean P in the preceding inter-session interval (i.e., between *t* – 1 and *t*) will be associated with lower social anxiety symptoms and social threat appraisals at the next assessment (*t*), on average. Conversely, when holding mean O and other controls constant, lower mean P will be associated with higher social anxiety symptoms and higher social threat appraisals at the next assessment, on average.

Hypotheses will be supported if both the following conditions are met; (1) the fixed effect coefficient for mean O is significantly different from zero and positive in sign and (2) the fixed effect coefficient for mean P is significantly different from zero and negative in sign (52). To determine whether the joint effect of these component variables on the outcome variable is more complex than a linear discrepancy effect, we will run a model including second-order terms (mean P^2^, mean P × mean O, mean O^2^) as predictors (47, 50, 53). Hypotheses will be supported if the simpler model fits the data better than the more complex model. Models will be run in R using the nlme or lmer package. We will use the maximum likelihood estimation method and compare model fit using likelihood-ratio tests.

We will separate within- and between-person information for all time-varying predictors using person-mean centering (90). In this method, the person-mean centered predictors are added to level one and their respective person-means are added to level two. The fixed effect coefficients for the person-mean centered predictors are estimates of purely within-person effects, without being confounded with between-person effects.

We will also consider whether time trends are present in the data by regressing social anxiety and exposure process variables on time in separate analyses. The presence of time trends in time-varying outcomes or predictors can potentially confound any observed relationships between these variables (56). In this study, treatment is designed to cause changes to the outcomes and predictors over time and these changes are of direct theoretical interest. In these circumstances, time de-trending is not recommended because it may artificially suppress the predictor coefficients of interest (90). To be conservative, if there are time trends in any of the predictors or outcomes, we will run a sensitivity test on the final models to determine whether the effects of interest remain after time detrending (by adding time as a covariate) (55, 90).

In principle, the data structure has a three-level nested structure where assessments are nested in participants who are nested in therapists. There may be some degree of correlation between participant assessments within-therapist (91). Therapist-related statistical dependency and therapist effects can be addressed using three-level models (assessments at level one, participants at level two, and therapists at level three). If each therapist in the study has at least two participants, then three-level models will be used (assessments at level one, participants at level two, and therapists at level three). Otherwise, two-level models will be used (assessments at level one and participants at level two) since it would not be possible to distinguish between therapist and participant variance (91). Some therapists may be more effective than others and it is possible (but not plausible) that therapists could moderate the effect of prediction error on the outcome. However, exploring therapist effects is beyond the scope of this study.

The secondary aim of the study is whether the average prediction error experienced during treatment predicts treatment outcome. We hypothesize that participants who experienced greater symptom improvements from baseline to 3-month follow-up will have experienced greater overall threat prediction error in their exposures during treatment. For each participant, we will calculate their mean P and O experienced across all exposures in treatment (termed txP and txO, respectively). The outcome will be symptoms at 16 time points from baseline to 3-month follow-up. We will examine the interaction of assessment point with txP and the interaction of assessment point with txO on the outcome. The hypothesis would be supported if both interactions were significant, and txO is positive in sign and txP is negative in sign. The secondary hypotheses will be exploratory because cross-level interactions likely require larger sample sizes to be adequately powered to detect a medium effect size (92).

The tertiary aim of the study is to explore whether prediction error relates to surprise and learning outcomes. We hypothesize that the larger prediction error (regardless of type) will predict larger feelings of surprise after an exposure and greater likelihood for participants to report a learning outcome. We will use the EMA data without aggregation to perform multi-level RSA with surprise as the outcome and P and O and their higher order terms as predictors. Response surface parameters will be examined to determine whether the hypothesis is supported (49, 50, 53).

We will test whether session modality (in-person vs. video teleconference) affects outcomes by adding it as a level one predictor to all final models. We retain it as a control variable if it improves model fit. We will check the internal consistency of the threat prediction and outcome measures (other exposure measures are single item) using multilevel modeling [see (93)]. We will check the validity of exposure process measures by looking for expected patterns of associations among these variables (94).

### Sample size

We used Arend and Shäfers’ (92) simulation-derived and case-sensitive guides to determine the sample size for adequate power in the primary analyses. We aim to recruit 65 participants so that for 7 observations per participant (data from sessions 5 to 12) in the primary analysis we could detect small-medium level one effects (standardized fixed-effect size = 0.18) with 0.8 power at a significance level of 0.05 and assuming a large intraclass coefficient of 0.5 (92). We plan to use full maximum likelihood estimation so that nested models that differ in fixed effects may be compared with likelihood ratio tests. The target sample size would be sufficient to provide accurate fixed effect estimates when using full maximum likelihood estimation, based on simulation-derived guidelines (95, 96).

If the actual sample size is low (less than 50), we will use restricted maximum likelihood estimation with small sample corrections so that estimation is unbiased (sample size as low as 15 can return accurate estimates with this method (96). In this case, for the primary analysis, we would focus on the core linear discrepancy model and conduct CRA. We would not be able to test whether complex or non-linear discrepancy models fit the data better than linear discrepancy effects because restricted maximum likelihood estimation doesn’t permit meaningful comparison of models that differ in fixed effects. Arend and Shäfers’(92) guidelines suggest that a medium level one effect could be detected with sample size as low as 30 people with 5 observations, 0.8 power at a 0.05 significance level, and large intraclass correlation (0.5).

Digital EMA is well accepted and feasible in psychiatric populations (97). However, missing data is expected and will be handled through maximum likelihood estimation, assuming it is missing at random (98).

### Progress

Recruitment began in 2019 and will aim to end by 2023. Thirty-one people have participated in the study as of January 2022. The original study protocol developed in 2019 and went through modifications due to the COVID-19 pandemic, technical issues, and unavoidable changes to clinic procedures. These changes are described below.

#### Changes due to the COVID-19 pandemic

The original study protocol specified in-person treatment only, as was typical for most CBT delivery before the COVID-19 pandemic. We modified the protocol in 2020 due to the COVID-19 pandemic and associated six lockdowns in Melbourne, which were among the longest in the world. The study authors suspended in-person treatment from 20/3/2020 to protect participants and comply with government regulations. Ethics approval was obtained on 8/4/2020 to continue treatment and data collection online through video teleconference and survey software, respectively.

Since then, local COVID-19 transmission and lockdown levels have fluctuated, and this will likely continue for the duration of recruitment.

#### Change in ecological momentary assessment software

We initially used the *SEMA3* application (99) for event-contingent EMA which runs on iOS (v8.0 +) or Android (v6.0 +) devices. In January 2021, an issue with SEMA3 was detected leading to partially complete surveys for some Android phone users. On investigation, the issue was caused by Android battery-saving measures where the SEMA3 app was turned off when running in the background. This meant that participants would complete the pre-experiment portion of the survey and then attempt their exposure, but in the meantime, SEMA3 was turned off by Android to save battery. This also prematurely closed the survey in SEMA3 and forced it to record as partially complete. The authors, in consultation with SEMA3 support, could not find a measure that would overcome this problem for Android users. We decided to change software to Qualtrics which we were using for other self-report assessments already.

We recreated the exposure process questionnaire with online survey software Qualtrics using the same content, text, conditional branching, and response types. A re-usable hyperlink to the survey was created. A researcher guides participants to place shortcuts to these hyperlinks on the home page of their smartphone screen. Participants may then tap this link and initiate a survey any time they attempt an exposure. Access to these Qualtrics surveys requires an internet connection, whereas SEMA3 can record responses offline. However, since web-browser apps are not impacted by battery-saving measures in Android, it allows participants to keep their exposure survey open for prolonged periods if needed. The authors decided that most smartphone users would be likely to have mobile data as part of their phone service, mitigating the drawback of not being able to provide offline responses.

#### Change in structured clinical interview

The original protocol used the Structured Clinical Interview for DSM-5, Clinical Version (100) for diagnostic assessment. In 2021, clinic leadership changed their procedures to use the DIAMOND instead. This decision was made to meet the clinical and business needs of the clinic vs. the needs of the current study. However, the DIAMOND has good psychometric properties for diagnosing mental disorders according to DSM-5 criteria (65).

## Discussion

This study will provide information about the frequency and type of prediction error experienced during exposure for SAD. It may help determine whether threat prediction error, as measured by self-report, predicts dynamic and long-term clinical outcomes in exposure for SAD. The study may also help to clarify the relationship between prediction error, surprise, and learning outcomes in the context of exposure. The above outcomes would contribute to evaluating the translation of extinction learning to exposure therapy.

The design has several limitations. Firstly, the results will be limited to SAD treatment and would need to be replicated in other anxiety disorder treatment. However, extinction is a basic learning process, so it is expected to operate the same way regardless of the foci of anxiety. Second, as a reviewer commented, it is possible that the EMA procedure itself may impact the exposure processes studied. Future research could explore different measurement methods to see whether it changes the results. Thirdly, the study is correlational, which may limit the potential for causal inference. It may be possible to conduct a randomized controlled trial comparing two exposure treatments that systematically differ in threat prediction error, while remaining the same in other aspects. Fourth, the results will be constrained to subjective (self-report) indices of prediction error. Subjective measures of threat prediction have evidence for being valid (101) and are immediately relevant to clinicians and patients (102). However, subjective measures of fear can diverge from physiological and behavioral measures, leading to debate as to whether they may reflect different neural systems (102–105). Further research using physiological markers of prediction error would complement and expand the proposed study. Fifth, as a reviewer commented, the current study does not explore potential moderators of exposure mechanisms, such as therapeutic alliance, patient self-efficacy, or patient capacity for distress tolerance. The current study also doesn’t explicitly explore the impact of prediction error on belief change or other outcomes. Future research will be needed to determine how other variables interact with prediction error and using a broader range of outcomes. Finally, replication in a large sample will be required to enable more sophisticated multivariate modeling that can account for more complex dynamic effects and control for measurement and sampling error (55).

This study will improve our understanding of exposure-based treatment for SAD and help determine whether extinction learning mechanisms translate to the clinic. Contemporary exposure therapy models are based on the proposition that prediction error is central to learning outcomes. Clarifying whether this proposition is true is thus important and will have important clinical implications.

## Ethics statement

The studies involving human participants were reviewed and approved by the University of Melbourne Human Research Ethics Committee, University of Melbourne, Melbourne, Victoria, Australia. The patients/participants provided their written informed consent to participate in this study.

## Author contributions

CW designed the study protocol under supervision from KF and LP and in consultation with PK. CW adapted the treatment manual with LP and KF supervising. LP, KF, and PK designed the parent project which was then modified in collaboration with CW to incorporate the current nested study. CW will manage the data collection and protocol implementation, and analyze final data under supervision by LP and KF and in consultation with PK. CW wrote the manuscript with feedback from KF, LP, and PK. PK advised on study design and statistical analysis. All authors contributed to the article and approved the submitted version.
