# Supplementary material for: Does prediction error during exposure relate to clinical outcomes in cognitive behavior therapy for social anxiety disorder? A study protocol
Source: Front Psychiatry. 2022 Nov 24;13:1000686. doi: 10.3389/fpsyt.2022.1000686 (PMC10111196; doi:10.3389/fpsyt.2022.1000686)
Supplement: Supplementary file 1 [file Data_Sheet_1.PDF]

Supplementary Material

1 Exposure Process Questionnaire

This document contains all three versions of the exposure process questionnaire as implemented in survey software Qualtrics (and previously in *SEMA3*). The standard version (EPQ) is shown in Table 2., the safety behaviors version (EPQ-SB) in Table 3., and video review version (EPQ-VR) in Table 4. The surveys were designed to be completed on smartphone devices. Each question is presented on a separate screen for ease of use and a progress bar is presented at the top of the screen. A forward and backward arrow buttons are presented at the bottom of each page to confirm the response and progress to the next page or go the previous page. *SEMA3* didn't have the functionality for backward cycling, so *SEMA3* surveys contained forward buttons only.

Table 2. EPQ

Item number	Item description	Displayed text	Item type	Displayed response options
1	Entry	This is the **Standard Behavioural Experiment** survey, used when doing behavioural experiments throughout treatment. If you chose this survey by mistake you can exit by choosing the relevant option below. In this survey, you will be asked to complete questions just before doing your planned behavioural experiment and then immediately afterwards. *Please wait until you are just about to do the behavioural experiment before starting the survey.*	Single choice	Ok, I'm ready to continue the survey!"<br><br>Actually, I don't want to continue with this survey right now (choosing this option will end the survey)
Conditional branching: end survey if "Actually, I don't want to continue with this survey right now (choosing this option will end the survey)" is selected for item 1.				
2*	Exposure description	What social activity will you do?	Open ended response	Typed text field

Item number	Item description	Displayed text	Item type	Displayed response options
3**	Exposure context	Where is the social activity taking place?	Single choice	During a treatment session<br><br>In between treatment sessions (for homework)
4*	Exposure novelty	Have you done this social activity yet in this treatment program?	Single choice	Yes, in a session or for homework<br><br>No, not yet
5*	Threat prediction description	What are your worst fears about doing this activity? Specifically, what are you afraid will happen?	Open ended response	Typed text field
6*	Threat prediction – worst fears	How strongly do you believe that your worst fears will happen during the activity?	Slider rating	Not at all (0) to Extremely (100)
7*	Threat prediction – judged negatively	How strongly do you believe that you will be judged negatively during the activity?	Slider rating	Not at all (0) to Extremely (100)
8*	Threat prediction – bad impression	How strongly do you believe that you will make a bad impression during the activity?	Slider rating	Not at all (0) to Extremely (100)
9*	Threat prediction – anxious appearance	How strongly do you believe that you will appear anxious during the activity?	Slider rating	Not at all (0) to Extremely (100)
10*	Threat prediction – how bad	How bad do you think the outcome of the activity will be?	Slider rating	Not at all (0) to Extremely (100)

Item number	Item description	Displayed text	Item type	Displayed response options
11*	Anxiety - pre	How anxious do you feel now?	Slider rating	Not at all (0) to Extremely (100)
12*	Mid-point	Ok. Now do the activity. Indicate below whether you completed the activity or not. Try to do this as soon as possible after completing the task so that it is fresh in your mind.	Single choice	Activity completed.<br>I didn't complete the activity.
Conditional branching: end the survey if "I didn't complete the activity" is selected for item 12.				
13*	Recency check	Are you completing this survey within 2 minutes of finishing the activity?	Single choice	Yes<br>No
Conditional branching: display item 14 if "No" is selected for item 13.				
14*	How recent?	Roughly how long ago did you finish the activity? *(Scroll down for all options)*	Single choice	Between 2 and 10 mins<br>Between 10 and 20 mins<br>Between 20 and 30 mins<br>Between 30 mins and 1 hour<br>Over an hour

Item number	Item description	Displayed text	Item type	Displayed response options
15*	Duration	Roughly how long were you in the activity for? *(Scroll down for all options)*	Single choice	Less than 5 mins<br>Between 5 and 10 mins<br>Between 10 and 15 mins<br>Between 15 and 30 mins<br>Between 30 mins and 1 hour<br>Over an hour
16*	Anxiety – post	How anxious do you feel now?	Slider rating	Not at all (0) to Extremely (100)
17*	Anxiety – peak	How anxious were you at your peak anxiety level during the activity?	Slider rating	Not at all (0) to Extremely (100)
18*	Anxiety - end	How anxious did you feel at the end of the activity?	Slider rating	Not at all (0) to Extremely (100)
19*	Surprise	How surprised do you feel about the outcome of the activity?	Slider rating	Not at all (0) to Extremely (100)
20*	Threat outcome – worst fears	How strongly do you believe that your worst fears happened during the activity?	Slider rating	Not at all (0) to Extremely (100)
21*	Threat outcome – judged negatively	How strongly do you believe that you were judged negatively during the activity?	Slider rating	Not at all (0) to Extremely (100)
22*	Threat outcome – bad impression	How strongly do you believe that you made a bad impression during the activity?	Slider rating	Not at all (0) to Extremely (100)

Item number	Item description	Displayed text	Item type	Displayed response options
23*	Threat outcome – anxious appearance	How strongly do you believe that you appeared anxious during the activity?	Slider rating	Not at all (0) to Extremely (100)
24*	Threat outcome – how bad	How bad do you think the outcome of the activity was?	Slider rating	Not at all (0) to Extremely (100)
25*	Safety behaviours use	How much did you use any safety behaviours to manage your anxiety during the activity? *Safety behaviours are things people do sometimes in social situations to manage their anxiety, such as avoiding eye contact, remaining very quiet, making an effort to get your words right, trying to not look anxious (e.g. trying not to shake sweat, blush) or rehearsing sentences in your mind.*	Slider rating	Not at all (0) to As much as possible (100)
26*	Interaction check	Were you interacting with someone during the activity? *Interacting means conversing with or doing an activity with one or more people. E.g. talking to someone, giving a presentation, being in a group conversation, buying something in a shop. An example of not interacting might be eating by yourself in public – i.e. even though you might have been seen by others, you didn't speak to anyone and weren't spoken to by anyone directly.*	Single choice	Yes<br>No

Conditional branching: display item 27 if "Yes" is selected for item 26.

Item number	Item description	Displayed text	Item type	Displayed response options
27*	Type of interaction partner(s)	Who were you interacting with? *(Select all that apply – scroll down for all the options)*	Multiple choices	Family member<br>Friend<br>Co-worker / class-mate<br>Romantic partner<br>Stranger<br>Acquaintance<br>Therapist<br>Other
28*	Location	Where did you do the activity? *(Select the option that best describes the location – scroll down for all the options)*	Single choice	Home<br>Family member's home<br>Public place<br>Psychology clinic<br>Work / school<br>Shop<br>Restaurant<br>Bar / Club / Pub<br>Party<br>Other
29*	Actual outcome	Compared to your initial predictions, what actually happened during the activity?	Open ended response	Typed text field

Item number	Item description	Displayed text	Item type	Displayed response options
30*	Learning check	Did you learn anything from doing this activity?	Single choice	Yes<br>No<br>Not sure
Conditional branching: display item 31 if "Yes" is selected for item 30.				
31*	Learning outcome	What did you learn?	Open ended response	Typed text field
End of survey				

**Core EPQ item shared across all three versions. **Only present in EPQ as this version may be completed in or between of sessions. Other EPQ versions are only completed in-session so this item was dropped on those versions for efficiency.*

Table 3. EPQ-SB

Item number	Item description	Displayed text	Item type	Displayed response options
1	Entry	This is the **Safety Behaviours Experiment** survey, completed in **Session 6** with your therapist. If you chose this survey by mistake you can exit by selecting the relevant option below.<br>In this survey, you will be asked to complete questions just before doing your planned behavioural experiment and then immediately afterwards. *Please wait until you are just about to do the behavioural experiment before starting the survey.*	Single choice	Ok, I'm ready to continue the survey!<br>Actually, I don't want to continue with this survey right now (choosing this option will end the survey)
Conditional branching: end survey if "Actually, I don't want to continue with this survey right now (choosing this option will end the survey)" is selected for item 1.				

Item number	Item description	Displayed text	Item type	Displayed response options
2*	Exposure description	What social activity will you do?	Open ended response	Typed text field
3*	Exposure novelty	Have you done this social activity yet in this treatment program?	Single choice	Yes, in a session or for homework.<br><br>No, not yet.
4	Safety behaviours description	What are your safety behaviours in this situation? *Safety behaviours are things people do sometimes in social situations to manage their anxiety, such as avoiding eye contact, remaining very quiet, making an effort to get your words right, trying to not look anxious (e.g. trying not to shake sweat, blush) or rehearsing sentences in your mind.*	Open ended response	Typed text field
5*	Threat prediction description	What are your worst fears about doing this activity? Specifically, what are you afraid will happen?	Open ended response	Typed text field
6*	Threat prediction – worst fears	How strongly do you believe that your worst fears will happen during the activity?	Slider rating	Not at all (0) to Extremely (100)
7*	Threat prediction – judged negatively	How strongly do you believe that you will be judged negatively during the activity?	Slider rating	Not at all (0) to Extremely (100)
8*	Threat prediction – bad impression	How strongly do you believe that you will make a bad impression during the activity?	Slider rating	Not at all (0) to Extremely (100)

Item number	Item description	Displayed text	Item type	Displayed response options
9*	Threat prediction – anxious appearance	How strongly do you believe that you will appear anxious during the activity?	Slider rating	Not at all (0) to Extremely (100)
10*	Threat prediction – how bad	How bad do you think the outcome of the activity will be?	Slider rating	Not at all (0) to Extremely (100)
11*	Anxiety - pre	How anxious do you feel now?	Slider rating	Not at all (0) to Extremely (100)
12*	Mid-point	Ok. Now do the activity. Indicate below whether you completed the activity or not. Try to do this as soon as possible after completing the task so that it is fresh in your mind.	Single choice	Activity completed.<br>I didn't complete the activity.
Conditional branching: end the survey if "I didn't complete the activity" is selected for item 12.				
13*	Recency check	Are you completing this survey within 2 minutes of finishing the activity?	Single choice	Yes<br>No
Conditional branching: display item 14 if "No" is selected for item 13.				
14*	How recent?	Roughly how long ago did you finish the activity? *(Scroll down for all options)*	Single choice	Between 2 and 10 mins<br>Between 10 and 20 mins<br>Between 20 and 30 mins<br>Between 30 mins and 1 hour<br>Over an hour

Item number	Item description	Displayed text	Item type	Displayed response options
15*	Duration	Roughly how long were you in the activity for? *(Scroll down for all options)*	Single choice	Less than 5 mins<br>Between 5 and 10 mins<br>Between 10 and 15 mins<br>Between 15 and 30 mins<br>Between 30 mins and 1 hour<br>Over an hour
16*	Anxiety - post	How anxious do you feel now?	Slider rating	Not at all (0) to Extremely (100)
17*	Anxiety - peak	How anxious were you at your peak anxiety level during the activity?	Slider rating	Not at all (0) to Extremely (100)
18*	Anxiety - end	How anxious did you feel at the end of the activity?	Slider rating	Not at all (0) to Extremely (100)
19*	Surprise	How surprised do you feel about the outcome of the activity?	Slider rating	Not at all (0) to Extremely (100)
20*	Threat outcome – worst fears	How strongly do you believe that your worst fears happened during the activity?	Slider rating	Not at all (0) to Extremely (100)
21*	Threat outcome – judged negatively	How strongly do you believe that you were judged negatively during the activity?	Slider rating	Not at all (0) to Extremely (100)
22*	Threat outcome – bad impression	How strongly do you believe that you made a bad impression during the activity?	Slider rating	Not at all (0) to Extremely (100)

Item number	Item description	Displayed text	Item type	Displayed response options
23*	Threat outcome – anxious appearance	How strongly do you believe that you appeared anxious during the activity?	Slider rating	Not at all (0) to Extremely (100)
24*	Threat outcome – how bad	How bad do you think the outcome of the activity was?	Slider rating	Not at all (0) to Extremely (100)
25	Safety behaviours experiment	How self-conscious did you feel?	Slider rating	Not at all (0) to Extremely (100)
26	Safety behaviours experiment	How good was your social performance?	Slider rating	Not at all (0) to Extremely (100)
27	Safety behaviours experiment	How much did you blush / shake / pause etc?	Slider rating	Not at all (0) to Extremely (100)
28*	Safety behaviours use	How much did you use any safety behaviours to manage your anxiety during the activity? *Safety behaviours are things people do sometimes in social situations to manage their anxiety, such as avoiding eye contact, remaining very quiet, making an effort to get your words right, trying to not look anxious (e.g. trying not to shake sweat, blush) or rehearsing sentences in your mind.*	Slider rating	Not at all (0) to As much as possible (100)

Item number	Item description	Displayed text	Item type	Displayed response options
29*	Interaction check	Were you interacting with someone during the activity? *Interacting means conversing with or doing an activity with one or more people. E.g. talking to someone, giving a presentation, being in a group conversation, buying something in a shop. An example of not interacting might be eating by yourself in public – i.e. even though you might have been seen by others, you didn't speak to anyone and weren't spoken to by anyone directly.*	Single choice	Yes<br>No
Conditional branching: display item 30 if "Yes" is selected for item 29.				
30*	Type of interaction partner(s)	Who were you interacting with? *(Select all that apply – scroll down for all the options)*	Multiple choices	Family member<br>Friend<br>Co-worker / class-mate<br>Romantic partner<br>Stranger<br>Acquaintance<br>Therapist<br>Other

Item number	Item description	Displayed text	Item type	Displayed response options
31*	Location	Where did you do the activity? *(Select the option that best describes the location – scroll down for all the options)*	Single choice	Home<br>Family member's home<br>Public place<br>Psychology clinic<br>Work / school<br>Shop<br>Restaurant<br>Bar / Club / Pub<br>Party<br>Other
32*	Actual outcome	Compared to your initial predictions, what actually happened during the activity?	Open ended response	Typed text field
33*	Learning check	Did you learn anything from doing this activity?	Single choice	Yes<br>No<br>Not sure
Conditional branching: display item 34 if "Yes" is selected for item 33.				
34*	Learning outcome	What did you learn?	Open ended response	Typed text field
End of survey				

**Core EPQ item shared across all three versions.*

Table 4. EPQ-VR

Item Number	Item description	Displayed text	Item type	Displayed response options
1	Entry	This is the **Feedback Experiment** survey, completed in **Session 7** with your therapist. If you chose this survey by mistake you can exit by choosing the relevant option below. In this survey, you will be asked to complete questions just before doing your planned behavioural experiment and then immediately afterwards. *Please wait until you are just about to do the behavioural experiment before starting the survey.*	Single choice	Ok, I'm ready to continue the survey!<br><br>Actually, I don't want to continue with this survey right now (choosing this option will end the survey)
Conditional branching: end survey if "Actually, I don't want to continue with this survey right now (choosing this option will end the survey)" is selected for item 1.				
2*	Exposure description	What social activity will you do?	Open ended response	Typed text field
3*	Exposure novelty	Have you done this social activity yet in this treatment program?	Single choice	Yes, in a session or for homework.<br><br>No, not yet.
4*	Threat prediction description	What are your worst fears about doing this activity? Specifically, what are you afraid will happen?	Open ended response	Typed text field
5*	Threat prediction – worst fears	How strongly do you believe that your worst fears will happen during the activity?	Slider rating	Not at all (0) to Extremely (100)
6*	Threat prediction – judged negatively	How strongly do you believe that you will be judged negatively during the activity?	Slider rating	Not at all (0) to Extremely (100)

Item Number	Item description	Displayed text	Item type	Displayed response options
7*	Threat prediction – bad impression	How strongly do you believe that you will make a bad impression during the activity?	Slider rating	Not at all (0) to Extremely (100)
8*	Threat prediction – anxious appearance	How strongly do you believe that you will appear anxious during the activity?	Slider rating	Not at all (0) to Extremely (100)
9*	Threat prediction – how bad	How bad do you think the outcome of the activity will be?	Slider rating	Not at all (0) to Extremely (100)
10	Video review experiment	How anxious will you feel?	Slider rating	Not at all (0) to Extremely (100)
11	Video review experiment	How anxious will you look?	Slider rating	Not at all (0) to Extremely (100)
12*	Anxiety - pre	How anxious do you feel now?	Slider rating	Not at all (0) to Extremely (100)
13*	Mid-point	Ok. Now do the activity. Indicate below whether you completed the activity or not. Try to do this as soon as possible after completing the task so that it is fresh in your mind.	Single choice	Activity completed.<br>I didn't complete the activity.

Conditional branching: end the survey if "I didn't complete the activity" is selected for item 13.

14*	Recency check	Are you completing this survey within 2 minutes of finishing the activity?	Single choice	Yes<br>No

Conditional branching: display item 15 if "No" is selected for item 14.

**Item Number**	**Item description**	**Displayed text**	**Item type**	**Displayed response options**
15*	How recent?	Roughly how long ago did you finish the activity? *(Scroll down for all options)*	Single choice	Between 2 and 10 mins<br>Between 10 and 20 mins<br>Between 20 and 30 mins<br>Between 30 mins and 1 hour<br>Over an hour
16*	Duration	Roughly how long were you in the activity for? *(Scroll down for all options)*	Single choice	Less than 5 mins<br>Between 5 and 10 mins<br>Between 10 and 15 mins<br>Between 15 and 30 mins<br>Between 30 mins and 1 hour<br>Over an hour
17*	Anxiety - post	How anxious do you feel now?	Slider rating	Not at all (0) to Extremely (100)
18*	Anxiety - peak	How anxious were you at your peak anxiety level during the activity?	Slider rating	Not at all (0) to Extremely (100)
19*	Anxiety - end	How anxious did you feel at the end of the activity?	Slider rating	Not at all (0) to Extremely (100)
20*	Surprise	How surprised do you feel about the outcome of the activity?	Slider rating	Not at all (0) to Extremely (100)

Item Number	Item description	Displayed text	Item type	Displayed response options
21*	Threat outcome – worst fears	How strongly do you believe that your worst fears happened during the activity?	Slider rating	Not at all (0) to Extremely (100)
22*	Threat outcome – judged negatively	How strongly do you believe that you were judged negatively during the activity?	Slider rating	Not at all (0) to Extremely (100)
23*	Threat outcome – bad impression	How strongly do you believe that you made a bad impression during the activity?	Slider rating	Not at all (0) to Extremely (100)
24*	Threat outcome – appeared anxious	How strongly do you believe that you appeared anxious during the activity?	Slider rating	Not at all (0) to Extremely (100)
25*	Threat outcome – how bad	How bad do you think the outcome of the activity was?	Slider rating	Not at all (0) to Extremely (100)
26	Video review experiment	How anxious did you look?	Slider rating	Not at all (0) to Extremely (100)

Item Number	Item description	Displayed text	Item type	Displayed response options
27	Video review experiment	Select the anxiety symptoms you displayed and rate their observability *(scroll down to see all the options)*<br><br>Blushed<br><br>Fidgeted<br><br>Stuttered<br><br>Shaky voice<br><br>Sweated<br><br>Long pauses (more than 5 seconds)<br><br>Trembled<br><br>Other	Multiple slider ratings	Not at all (0) to Extremely (100)<br><br>*One slider for each anxiety symptom presented on the same page*
28*	Safety behaviours use	How much did you use any safety behaviours to manage your anxiety during the activity? *Safety behaviours are things people do sometimes in social situations to manage their anxiety, such as avoiding eye contact, remaining very quiet, making an effort to get your words right, trying to not look anxious (e.g. trying not to shake sweat, blush) or rehearsing sentences in your mind.*	Slider rating	Not at all (0) to As much as possible (100)

Item Number	Item description	Displayed text	Item type	Displayed response options
29*	Interaction check	Were you interacting with someone during the activity? *Interacting means conversing with or doing an activity with one or more people. E.g. talking to someone, giving a presentation, being in a group conversation, buying something in a shop. An example of not interacting might be eating by yourself in public – i.e. even though you might have been seen by others, you didn't speak to anyone and weren't spoken to by anyone directly.*	Single choice	Yes<br>No
Conditional branching: display item 30 if "Yes" is selected for item 29.				
30*	Type of interaction partner(s)	Who were you interacting with? *(Select all that apply – scroll down for all the options)*	Multiple choices	Family member<br>Friend<br>Co-worker / class-mate<br>Romantic partner<br>Stranger<br>Acquaintance<br>Therapist<br>Other

Item Number	Item description	Displayed text	Item type	Displayed response options
31*	Location	Where did you do the activity? *(Select the option that best describes the location – scroll down for all the options)*	Single choice	Home<br>Family member's home<br>Public place<br>Psychology clinic<br>Work / school<br>Shop<br>Restaurant<br>Bar / Club / Pub<br>Party<br>Other
32*	Actual outcome	Compared to your initial predictions, what actually happened during the activity?	Open ended response	Typed text field
33*	Learning check	Did you learn anything from doing this activity?	Single choice	Yes<br>No<br>Not sure
Conditional branching: display item 34 if "Yes" is selected for item 33.				
34*	Learning outcome	What did you learn?	Open ended response	Typed text field
End of survey				

Core EPQ item shared across all three versions.
